# Reports on Cities and Towns

**Published:** 1855

**Authors:** 


### SANITARY AND SOCIAL SCIENCE.

### REPORTS ON CITIES AND TOWNS.

### I. Glasgow.—*Population*, 396,000.

*Mortality.*—During the past seven years, the deaths in Glasgow (including two visitations of cholera) have numbered 86,924, or as many persons as constituted the whole population in the year 1775. Of these, 16,356 occurred during 1854. The infantile mortality of the seven years was, in proportion to the whole mortality, 44·56 per cent.

*Diseases.*—The most striking, because the most exceptional, is the cholera, which, with its attendant diarrhœa, cut off in 1854 not fewer than 4,612 inhabitants. In the three visitations of this disease—viz., in 1832, 1848–49, and 1853–54—it exhibited some peculiarities. In the first, its ravages were almost limited to the destitute: in the second, it attacked the better parts of the city: in the third, it shewed itself chiefly in the northern and eastern districts, and after June it was found in every quarter, and at last it took its victims from the best parts of the city.

*Children's Diseases* were prevalent in 1854. There dies in Glasgow annually, on an average, by measles, 1 in 795; by hooping-cough, 1 in 539; by scarlet-fever, 1 in 711; by croup, 1 in 2,118.

*Small-pox.*—Vaccination is much neglected; 467 died in Glasgow and its suburbs from small-pox in 1854.

*Consumption* has carried off 14,933 in the past seven years; 2,350 in 1854.

*Burial Places.*—Urban and cryptal burials still continue in Glasgow, and many of the graveyards are in the most thickly populated parts of the city. This form of burial is, however, decreasing; and there exist four cemeteries out of the city, called respectively the Sighthill, the Eastern and Southern Necropolis, the Dalbeth, and the Necropolis. In these, the number of burials in 1854 have increased no less than 1,894 over those of the previous year. The pauper burials in 1854 amounted to 3,564.

*Drownings in the Clyde.*—Out of 161 cases of accidents and attempts at suicide in 1854, 123 were saved and 38 were drowned. There were drowned in canals, &c., 9; and 18 persons died in the streets.

*Births and Marriages.*—In 1854, 8,735 children were baptised; but there must be, on an average, an annual number of births amounting to 13,000—which, indeed, are not sufficient to replace the last year's waste of life; so that Glasgow would be retrogressing in population but for the constant flood of immigration. The marriages in 1854 were 4,662.

*Aliments: Animal Food.*—There were slaughtered in Glasgow during 1854—Oxen, 27,881; calves, 2,004; sheep, 94,027; lambs, 44,098; goats, 36; pigs, 4,633. Calculating for the animal food imported into the city, it is estimated that there are annually consumed in Glasgow not less than 45,000,000 lbs. weight of fresh and salted meat (a total which gives an average of about 113 lbs. to each person), 144,000,000 lbs. of bread, 3,367 tons of fish, 1,100 tons of cheese, 918 tons of onions, and 1,075,536 lbs. of fruits.

*Spirits.*—Beer and porter are not much drunken here. There is an annual consumption of about 250,000 gallons of foreign and colonial spirits; and, assuming that Glasgow receives an equal share with Scotland generally, it follows that the annual consumption of whiskey is about 950,000 gallons at proof. Of tea, the consumption in 1854 was 3,008,208 lbs.; of sugar, 48,559 tons; of molasses, 25,020 tons.

The *Houses and Places of Business* number 80,304: or of dwelling-houses, occupied, 68,916; unoccupied, 1,285; of places of business, occupied, 9,754; unoccupied, 349.

The *Water* supply of the city and its suburbs is not less than 15,300,000 gallons per day, giving, with deductions, an average of nearly 39 gallons to each inhabitant. This enormous amount is expended mainly in plunge-baths, of which there are no fewer than 6,638, and water-closets 12,114. The water is supplied from the Clyde by two companies, the Glasgow Water Company and the Gorbals Company. If the water is not of the purest quality, it is distributed more abundantly than in any other city in the world.

*Gas* was consumed in 1854 to the extent of 589,000,000 cubic feet.

*Police Crime.*—12,218 men and 3,559 women were brought before the magistrates in 1854 for various crimes. Of these 10,459 were cases of drunkenness. The evil effects of punishment for the suppression of juvenile crime are most obvious in this city.

*Charitable Institutions* are abundant in Glasgow. There are two Houses of Refuge, one for boys, the other for girls. Into the former, 36 were admitted in 1854. The results of both are highly encouraging. In a second female charity, called the Shelter, there are 40 women. In the industrial and ragged schools, there are 274 boys and 141 girls. In the Model Lodging-house, 118 inmates, on the average, are admitted nightly. In the House of the Houseless, 75 are nightly admitted. In the House of Industry, there were 50 applicants admitted during the year.

*Pauperism* in Glasgow and the suburbs is very great. 5,576 casual poor received relief in 1854.

The rational *Amusements* for the working classes are the Andersonian University Museum, the Royal Botanic Gardens, and the Diorama.

(*Compiled from* Dr. Strang's "*Social and Economic Statistics of Glasgow*, 1854.")

### II. Swansea.—*Population*, 23,890.

The two following reports on the sanitary conditions and mortality of Swansea and Bedford, have been drawn up specially for The Journal of Public Health:—

The borough of Swansea, as defined by the Public Health Act, consists of portions of the parish of Swansea, and the parish of St. John, portions also of the parishes of Llangavelach and Llansamlet, these forming three separate registration districts. From this circumstance much difficulty arises in rightly estimating the statistics of various districts under the Public Health Act, and shews the necessity of a simultaneous action and extent between registration districts and districts under the Public Health Act. To illustrate this, and shew the difficulties that may arise, it may be sufficient to state that in the parish of Swansea, and the registration district of Swansea, 2,227 persons lived beyond the boundaries of the borough; the mortality, therefore, of these persons must be subtracted from the returns of the Registrar-General before a correct estimate of the mortality of the town of Swansea can be arrived at. Leaving out the parts of the parishes of Llansamlet and Llangavelach, within the borough of Swansea, we shall in the present paper give only a short abstract of the mortality of the town-proper. Rising gradually from the beach the town is built on the eastern and western sides of the river Tawe, at a considerable height above high-water mark on the western bank, on which the main part of the town is built, and extending from the sea beach on the south to the more elevated part on the north. Built on a loamy, gravelly soil, covering to an average depth of seven feet a bed of bluish clay, the town is placed at the limit of the coal measures on their southern crop. "From the manner in which the more porous sandstone and other beds are mingled with those comparatively impervious to the passage of water"* abundant springs of water are found in the vicinity of the town. The lower level of the town is supplied by the Bryn Mill reservoir,† but the upper parts being above its level, are left to the chance supply of springs, wells, &c.,--a supply very inadequate to the wants of a dense population, and undeniably the cause of great sickness, distress, and mortality. When cholera visited this town in 1830, the lower portions, then without a waterwork supply, were almost exclusively the seat of the epidemic; but in 1849 this was entirely changed, the supply of water almost marking out the limit of extension of the disease, and the parts beyond adequate and pure water supply, being the ones in which the epidemic expended its virulence; for it must be borne in mind, that the very soil which allows a supply of water from wells, also in the absence of proper sewerage and drainage, allows the percolation of the more liquid portions of sewage matter from cesspools, one of which must be of necessity attached to every house where closet accommodation has no provided drainage outlet. From this fact, there arises a great increase of mortality in streets, one portion of which is at a level considerably exceeding another, thus allowing a ready filtration through the soil. The mortality from this cause would, indeed, be much greater and more alarming, did not the porosity of the soil, which allows so free passage, also act as a filtered disinfectant and materially diminish the otherwise poisonous character of the matters in their transit. At the last census the town-proper of Swansea numbered 23,890 inhabitants, of whom 11,420 were males, and 12,470 females, living in 4473 houses, or 5·34 individuals to eaeh house, this being considerably below the average number of large towns, and materially contributing to diminish the mortality, otherwise incident on deficient sanatory regulations Each house, as a rule, contains but one family, through the prevalence of the cottage system, everywhere to be found in the vicinity of such large copper and other ore smelting works as are located at and in the neighbourhood of Swansea. The mean

* Report of Sir H. de la Beche.

† Hardness of water 8° 7′;
Quantity of matter per imperial gallon, 9·20.

total mortality of the town is 560 per annum, or 2·34 per cent. per annum, of these in 1854 a large proportion, so much as 40 per cent., occur under five years of age, and this mainly from convulsions or diseases of the air-passages, caused to a great extent from want of the slightest care in the preservation of health or attempt to ward off the progress of disease. Owing to most of the population—a very mixed one of Welsh, Irish, and natives of the south-western counties of England—being engaged in the copper works and collieries, to each of which a surgeon is attached, no population could be more freely supplied with assistance, but inattention and exposure are ill compensated for by the soundest and best advice. Medical aid is always at hand, but is too often sought when too late to be of any avail to ward off a fatal issue to the attacks of bronchitis, pneumonia, croup, and convulsions, which carry off the larger proportion of children dying under five years of age. Epidemics are extremely mild,—scarlatina only averaging two per cent. of deaths in the whole borough; and in the year 1853, *only one death* occurring from this cause. In one part of the borough, scarlet fever, in a very fatal form, assuming a low remittent type, prevailed in the autumn of 1849, but did not extend to the town. It will be found that, in many districts of England, the same epidemic followed cholera at the same period; and it would be highly instructive, could a body of accurate observers mark by dates the lines of these epidemic visitations of disease over the whole of England and Wales, marking particularly the influence not only of surface but deep soil, water supply, &c., in determining the severity and continuance of the disease. The invasion of epidemics and their decline is well marked in the last visitation of small-pox to the town of Swansea: thus we find—

| | |
|---|---|
| In 1849 . . . | no case. |
| „ 1850 . . . | 1 „ |
| „ 1851 . . . | 2 cases. |
| „ 1852 . . . | 104 „ |
| „ 1853 . . . | 10 „ |
| „ 1854 . . . | no case. |

Of this large mortality, in nearly every instance,—only two being, I believe, exceptions,—the persons had been *unvaccinated;* and where, in a family, one member had not adopted this preservative, the disease manifested itself, either entirely sparing every other inmate, or assuming so mild a form as to be attended by none of the symptomatic fever, and only manifesting itself in a few—often not more than two or three—scattered pustules.

Measles, again, pursues the same course: thus 1 is the average mortality or this disease, except in 1851, when the mortality rose to 31; but, in every instance, this mortality is caused rather from the sequelæ, pneumonia, bronchitis, &c,, than from the severity of the primary attack or its accompanying fever; careless exposure to cold air being the most frequent cause of mortality, the disease itself, in almost every case, assuming a very mild form, and rapidly yielding to ordinary remedies. Typhus has a mortality only of 1 in 280 of the whole number of deaths, while continued fever amounts to 4 per cent. of the whole deaths,—this generally assuming a gastric type, of the typhoid character, but not usually associated with ulceration of the intestinal glands; true typhoid fever only averaging 1 in 560 of the whole number of deaths. Hooping-cough, which assumes here the true epidemic character, is rarely fatal, except, as in measles, when accompanied by pneumonia or convulsions. Of all the deaths recorded, 1 in 9·4 are returned as occurring from phthisis; but from this considerable deductions must be made for children under five years of age, who, having received no medical attendance, are, for want of a better name, returned by the friends as dying of decline. From a careful analysis of all deaths from phthisis in the town, 1 in 10·27 may be considered as a more correct estimate.*

* The total deaths from this cause above five years of age being—

2·92 Males<br>

25·4 Females<br>

——<br>

54·6 Total } per annum; or { 1 in 390 Males. 1 in 490 Females.

As a general summary of this short abstract of the mortality of the town of Swansea, it may be observed that much unnecessary mortality occurs for want of adequate drainage, sewerage, and water supply; that chest diseases, especially in children, prevail, but are ordinarily easily amenable to treatment; that epidemic diseases universally appear in a very mild form, rarely proving fatal, except in their secondary manifestations; and that no peculiarities of disease arise from the industrial occupations of the labouring classes (chiefly colliers and copper smelters), except the affections incident to those who are exposed to great vicissitudes of artificial temperature.

W. H. MICHAEL.

*(To be continued.)*

---

### III. Bedford.—*Population*, 13,000.

The town of Bedford stands upon the river Ouse, in what is called the Valley of the Ouse, with a range of hills north and south, at some distance from the town. The neighbourhood is comparatively open in the direction of east and west.

Geologically, it stands upon an exposed surface (excepting the valley gravel deposits) of the upper part of the lower oolite, surrounded by an amphitheatre (as it were) of the Oxford clay. These oolite rocks have never been defined, probably they are members of the cornbrash.

The population of the town is between 12,000 and 13,000, principally agricultural. The manufacture of ploughs and harrows is carried on to some considerable extent.

Its latitude is 52·8·0 N., longitude 0·1·51 W.
Height above the level of the sea about 100 feet.

The following are the results of meteorological observations taken in Bedford during the last four years, viz.:—

Mean reading of barometer, 29·817 in.
Range of barometer, 1:064 in.
Mean temperature, 48°.8
Range of temperature, 15°.5
Mean degree of humidity, 0·811.
Mean annual amount of rain, 22·63 in.

The mortality per thousand of the inhabitants for sixteen years, inclusive of deaths in the public institutions, was 27·1; the mortality per thousand for seven years, as reported by the Registrar-General to the Board of Health, was 28·0.

This high rate of mortality induced more than one-tenth of the rate-payers to petition the Board of Health for an investigation of the causes of such mortality. In compliance with this requisition, the Superintending-Inspector William Lee, Esq., carefully inspected the town, and received evidence from the inhabitants in the summer of 1854. Mr. Lee's Report has not yet been published, but is daily expected. In the absence of this Report, the following facts may be relied upon, viz.:—

That the mortality of Bedford is high in comparison with many other towns, not more favourably situated.

That the annual mortality in proportion to the population, is much greater than in the surrounding rural districts.

That the proportion of deaths in early childhood to the total mortality is greater than in the rural districts.

That the proportion of deaths, above the age of sixty years, is much less than in the rural districts.

That the proportion of deaths between the ages of thirty and sixty years, is much larger than in the rural districts.

That the proportion of deaths from the zymotic class of diseases, is considerably higher than in the rural districts.

We have no strong grounds of complaint against the natural circumstances and situation of the town. It is true that it would have been better had our ancestors seen the advantages of the slope on the north as a site for the town; but as it is, Bedford is not *naturally* more unfavourably situated for drainage and consequent dryness of the soil and the atmosphere, than many other towns which have a far higher sanitary character. The river *naturally* has a considerable fall at Bedford, and *ought* to run at a lively pace past the town, as, without doubt it did in old times, when the monks selected Newnham as a pleasant site for an abbey. Let it be noticed, however, that while we acquit the *natural* river Ouse of blame on the score of the mortality in Bedford, we can by no means pronounce the same favourable verdict on the actual river, or rather say, mass of half stagnant water, which now stretches itself—not flowing but only lazily wallowing to and fro—between our bridge and the site of the old mill at Newnham. Nothing really satisfactory can be done for the drainage and general purification of Bedford, while a large body of water in a great measure stopped in its course, extends through the centre of the town.

It has been proved by facts too numerous to leave any room for doubt that, —*cœteris paribus*—the rate of mortality is highest (and of course disease is most prevalent) in those towns and those parts of towns where deficiency of drainage, ventilation, and other necessary means of health are observed. In the deficient drainage and ventilation, the deficient supply of pure water, the dirty and crowded courts, cesspools, pig-styes, open ditches, slaughter-houses, unpaved streets, &c., we find the special causes of the extra rates of mortality in this town. Sixty deaths from fever have occurred in the Bedford parishes within one year; and any one who has inspected our worst localities—Gravel Lane, Allhallows Lane, White Horse Street, Beauchamp Row, Hawes Street, Diamond Alley, Waterloo, Pepper Alley, Bell Court, &c.,—need not wonder at the spread of fever or the inroads of cholera in such places. Several of these localities the cholera chose, with unerring instinct, in the years 1832 and 1849. The drainage of the town is very defective. Our largest main sewer passes down Gwyn Street; along the High Street, the most important street in the town, there is no main sewer, and the drainage is very imperfect. House draining, except for dwellings of the better class, is generally very deficient throughout Bedford, and street drainage is equally defective. It has been proved that cesspools and dung-pits are a great source of the contamination of water of neighbouring springs; and it is estimated that there are not fewer than 3,000 sewers in this little town. Water percolating through the stagnant dung-pits, often permeates the soil, and then drains into wells and springs. In this way, too, more than in the deterioration of the atmosphere, are intramural burial-grounds so prejudicial to public health. Cesspools, and similar nuisances, close to houses, are found in all quarters of our town. Many of our streets are so ill pitched and flagged, that pools of water abound in rainy weather, and mud makes some parts almost impassable in long-continued rain. Many of the cottages, even of recent construction, are ill arranged for ventilation. Some of them are built back to back. An arrangement too commonly found is the entrance to a bed-room with a window close to the entrance, but no fire-place, so that the room is a *cul de sac* with no possibility of a thorough ventilation, even when the window is opened. Slaughter-houses, with the most offensive stench, and pig-styes, must also be noticed amongst our nuisances.

On the whole, we are compelled to say that Bedford is in a very filthy condition; that its sewerage and drainage are not sufficient for the health of the people; that cesspools and other receptacles of refuse are allowed to remain as very serious nuisances; that the surface of many streets is left in a very dirty state; that many of the courts and alleys are close and narrow; that numerous cottages have been so built as to be deprived of sufficient light and ventilation: and that the supply of pure water is deficient in several parts of the town.

The streets, generally, are macadamised; the footpaths are pitched, a part of them in the High Street, consisting of flag-stones. The roofing of the houses is mostly of slate or tiles; and the town is lighted with gas. During the cholera epidemic of last year, Bedford escaped, not a singe case of that disease was known to occur in the town. The "Sanitary Committee" of the Board of Guardians, and the "Improvement Commissioners" had been exceedingly active all the year. Many hundreds of nuisances had been removed, and the town had been placed in as effective a sanitary condition as was possible by the limited powers of these public bodies. To this fact was, most probably, due the late exemption of the town from cholera. The "Sanitary Committee" of the Board of Guardians published a valuable Report of these proceedings, which bears ample testimony to their very praiseworthy, active, and successful efforts. Until the town is radically improved in a sanitary point of view, there will be large scope for their continued exertions, and afterwards for their vigilant surveillance.

Vaccination is efficiently practised in the town and neighbourhood of Bedford. In the entire district of the Bedford Union, comprising a population 36,000, the number of births in one year were 1083. The number of children successfully vaccinated under one year of age were 764; above one year of age, were 2136. Total number of children successfully vaccinated, 2900. Although so many as 2900 persons in this district were successfully vaccinated during the year, only 509 cases of *successful vaccination were registered.* In one sub-district in which 108 persons were successfully vaccinated, only nine cases were registered. This deficiency in the registration of successful cases arises from the fault of the *medical* "Public Vaccination Act."

An excellent cemetery is being erected at a distance on the northern side of the town, and an order has been received for closing the burial-grounds within the town after a certain date. If the town of Bedford possessed a character for healthiness, it would become one of the most flourishing places in the kingdom. Through the munificence of Sir Wm. Harpur, Lord Mayor of London during the sixteenth century, Bedford possesses one of the finest educational establishments in England. The proceeds of an estate, amounting to some £12,000 annually, are expended in the gratuitous education of the children of the inhabitants, with the exception of a share which is distributed in almshouses, marriage portions to maidens, to the apprenticing of poor children, &c. It is evident that in Bedford there exists a very special inducement for procuring the best possible sanitary condition. Upon this, in fact, must mainly depend its future prosperity.

HERBERT BARKER, M.D.

Bedford, March 10th, 1855.

---

### LAST REPORT ON THE SANITARY CONDITION OF THE EASTERN ARMY.

Dr. Hall, in his report to Lord Raglan, bearing date March 2nd, expresses a confident hope that a favourable change has taken place in the fever at Balaklava, from low typhoid to that of remittent, or even intermittent form.

A convalescent establishment of huts has been erected, overlooking the sea, in which from 400 to 500 men can be placed, instead of being sent to Scutari. These huts are on a dry ridge of land, removed from the noise and stench of the town, not too far from the stores, and near to a running stream. Out of 442 patients treated at the general hospital, only three casualties (deaths) occurred. The huts in the vineyard in front of the hospital have prevented the necessity of overcrowding it. Its ventilation has been improved by boring two rows of large augur holes through the wooden ceiling of the wards. The sick have the benefit of able nurses. The Cavalry Division, the Artillery, and the Highland

Brigade, seem to have enjoyed tolerably good health. The Second, Fourth, and Light Divisions, have improved in health; but the Third, and the Brigade of Guards, continue sickly. Dr. Hall has instructed the medical officers to inspect the men for scurvy and skin diseases, and for seeing that the men are clean in their persons, and change their shirts and flannels at stated periods. In the Fourth Division, 138 out of 2,596 men inspected shewed signs of scurvy. If the issue of fresh meat, vegetables, and lime-juice, could be insured, now that the men are warmly clad and sheltered, a manifest improvement would soon take place in their health, especially if a greater exemption from duty could be allowed. Dr. Hall concludes by saying that he believes the greatest difficulties are over, and as the winter is nearly at an end, he is full of hope and confidence.

---

### XENOPHON'S OPINIONS ON THE SANITARY MANAGEMENT OF ARMIES.

In his work on the Education of Cyrus (*Cyropædia*, book I.)' Xenophon introduces a dialogue between Cyrus and his father, Cambyses, on the health of armies, of which the following are interesting translated extracts:

Cambyses is supposed to be giving instruction to his son, as to the management of the army, which is about to be sent to the aid of Cyaxares, king of the Medes.

" *Cambyses.*—But, my son, there are certain occurrences in which we have not to contend with men, but with circumstances over which it is not easy to prevail. In the first place, as thou art aware, unless thy army is provided with necessaries, thy power will speedily come to nought.

" *Cyrus.*—But, my father, Cyaxares says that he will provide for all who set out, how many soever they be.

" *Cambyses.*—Wilt thou then proceed on thy expedition, relying on the resources of Cyaxares?

" *Cyrus.*—Certainly.

" *Cambyses.*—What then, dost thou know the extent of his resources?

" *Cyrus.*—Not all, indeed.

" *Cambyses.*—And dost thou rely on such uncertainties? Dost thou not foresee that thou wilt have need of many things, and that thou must of necessity incur further expenses?

" *Cyrus.*—I perceive it.

" *Cambyses.*—If, then, the resources of Cyaxares fail, or if possessing them he does not supply them, in what condition will thy army be?

" *Cyrus.*—Certainly bad. But do thou, my father, if thou perceivest any way in which I can promote the supplies of necessaries, explain it to me while we are in a peaceful country.

"*Cambyses.*—Dost thou ask in what way thou canst provide for the wants of the army? On no one does the duty of supplying necessaries devolve more than on him who commands the armies. Thou oughtest to consult with Cyaxares, lest any of the things which should be at hand should fail you. But chiefly of all, bear in mind never to put off the provision of necessaries until the moment comes when they are required. But when thou hast most abundant resources, then provide against want; for when thou appearest not to be destitute, thou wilt gain more of those from whom thou askest, and wilt be free from blame with thy soldiers. And by this way thou wilt gain from them more respect; and if thou desirest to aid or to injure any with thy power, thy men will serve thee more efficiently if they are well cared for; and, believe me, thou wilt be more able to use most persuasive argument when thou shewest that thou art able both to do good and to injure."

Cyrus then proceeds to relate a former dialogue he had had with his father, on the occasion of requesting from him some pecuniary supplies for the army, in the course of which he says:—

"*Cyrus.*—In giving me the supplies of money thou didst interrogate me in this manner,—'Does the man to whom thou art about to carry this money, make any mention to thee of the domestic affairs of the army? for soldiers require the necessaries of life no less than do servants at home.' And when I replied that he had said nothing on this subject, thou didst further inquire whether he had ever spoken to me regarding the health and strength of the army; these being subjects about which a general should be anxious, as well as about his immediate military duties . . . . At last thou didst ask me, what instruction he professed to give me in the management of an army? To this I replied,—in military tactics. And then thou didst laugh, and, going through each subject, didst shew that the art of tactics was of no use to the soldiers, unless they were supplied with the necessaries of life, and in good health. . . . . With regard to food, I am sufficiently convinced that Cyaxares will provide us therewith. With regard to health, since I have heard and seen that those states which seek for good health educate physicians, and that commanders take with them physicians for the sake of the soldiers,—I, too, therefore, as soon as the present expedition was intrusted to me, gave my attention to this subject, and I think that I have with me very competent medical men.

"*Cambyses.*—But these physicians, my son, of whom thou speakest, are like menders of torn garments, and thus they cure those who have fallen sick. But thy chief anxiety should be to provide for health; for thou oughtest to take care to prevent the army from falling into sickness at all.

"*Cyrus.*—And how shall I accomplish this object?

"*Cambyses.*—If thou art about to remain long in a place, thou must first take care to pitch thy camp in a healthy spot, for men never cease to speak of unhealthy places as well as of healthy; for the bodies and the faces of men are signs of both."

Our modern rulers and commanders might, we think, take a few useful hints from the above-named precepts of the great soldier, historian, and philosopher of antiquity.

---

### TRADES WHICH AFFECT THE EYES.

In the course of last year, a committee on industrial pathology, appointed by the Society of Arts, issued the subjoined series of queries on the injuries resulting from handicraft work to the eye:—

"1. What occupation have you known to injure the eyes; by exposing them to chips, splinters, dust, grit, or fluff; to chemical fumes; to a glare or deficiency of light; to excessive heat or cold; by overstraining the organs; by indirect influence?

"2. What particular part of such operations are hurtful; and in what manner does the baneful cause operate?

"3. Does the evil apply to one sex, age, or kind of persons more than another?

"4. Can you point out any contrivance or arrangement which has been found useful in guarding against injury, or any medical treatment which has proved successful in mitigating its effects; or can you suggest any new means for prevention or relief?

"5. Can you point out any contrivance for facilitating or guiding the work of the needle, with a view to diminishing the distress of the eyes?

"6. Can you suggest any improvements in artificial light with respect to cheapness, quality, and convenience, for the use of persons engaged in minute work, either singly or many together?"

On the answers returned to these queries, the committee founded an interesting and valuable report, which is published in the *Journal of the Society of Arts* for January 12, 1855. It is signed by Dr. T. King Chambers, Mr. Simon, F.R.S., and Mr. T. Twining, jun. The conclusions drawn from the evidence given are the following:—

"1. The following classes of artisans are exposed to injury of the eyes from chips, splinters, dust, grit, or fluff, viz., engineers, masons, stonecutters, stonebreakers, bricklayers, soda water bottlers, turners, fitters, hammermen and smiths, cutlers, railway guards, rock blasters and quarrymen, millers, chimney-sweeps, workers in cotton, flax-dressers, feather-cleaners, drug grinders (especially in grinding blistering flies), shoemakers (from breaking of the awl); and the following appliances have been found useful in preventing the ill consequences of such exposure, viz., for those liable to blows from large portions of hard substances, such as stonebreakers, &c., coarse metal netting as eye-guards; and for those exposed to the finer dust, crape-spectacles, while at the same time free ventilation of the apartments they work in would relieve much of the inconvenience.

"2. The following suffer from the chemical nature of the substances which, in the shape of solid particles, get under the eyelids, viz., bricklayers, workers in lime, and workers in potash. No special preventive seems to be here pointed out, beyond the placing within reach of the workmen the ready means of immediately cleansing the parts with pure water. Some such apparatus as that described in Mr. White Cooper's communication might be placed in the workshop or superintendent's office. The action of chemical fumes, strictly so-called, has not been reported to cause injury.

[Mr. White Cooper's apparatus is thus described by him in his report :—

"Dust and Flue.—A simple and efficacious mode of cleansing the eyes would be by having suspended, in cotton and other manufactories, douches for the use of the workpeople. A cistern, capable of holding three or four gallons of water, is fastened to the wall; it is open at the top, to admit of its being filled, and closed by a well-fitted lid, to exclude impurities; a funnel-shaped zinc tube, furnished with a fine strainer, is attached to the bottom. From this extends a pipe of quarter-inch bore; the end is curved, and furnished with a stop-cock, and a rose is attached; the pipe leads into a zinc basin, furnished with an ordinary plug and waste-pipe, into which the water falls. By simply holding the face on the rose, and turning the cock, more or less, a regulated jet of water can be thrown upon the eyelids, which would effectually remove all impurities, and afford the eyes a most refreshing bath. Mr. Wilde, of Dublin, was the first to recommend this description of douches for hospitals."]

"3. The following suffer from excess of light or glare proceeding from the material used, viz., furnace men, gilders, and bookbinders. No practical remedy for this inconvenience has been suggested, as spectacles, which intercept the light, would diminish the efficiency of the workman. It may be observed that there is a great difference between excessive illumination of the work, and excess of light on the eye. The latter is the most common, and is considered under a separate head.

"4. The following suffer from deficiency of light, viz., dressmakers, tailors, sempstresses, coblers, and, in fact, all who, having to direct the needle to a definite spot, are unable to command the requisite amount of direct illumination.

"5. The ill effects of deficiency of light are much aggravated by working long on the same material or colour. The remedies for this and the foregoing evil are, increase of light and variety of work.

"6. Flickering of light is a great evil, which is felt much by compositors, and all who work at minute objects by gas illumination. The simple remedy for this is the employment of glass chimneys.

"7. It seems improper that an equal quantity of artificial light should fall on the work and on the eyes of the workman. If that is the case, the latter become overstrained. This evil, when it occurs, is easily obviated by shades to the light, which defend the eye, and throw the illumination on the required object. The shades should be made of white or light coloured material, so as to reflect as much light as possible. Ground glass between the light and the worker is injurious, by intercepting and diffusing the illumination instead of directing it on to the object.

"8. It seems doubtful whether heat and cold have much ill influences over the healthy eye; but when it is in a weak irritated condition, there is no doubt but that they are injurious.

"9. Bad ventilation, constrained postures, over-indulgence in spirituous liquors, the fumes of tobacco, and all other violations of healthy habits, are injurious to the eyes at the same time as to the rest of the body, and aggravate the bad effects of the above-named industrial occupations.

"10. The employment of the eye when the body is in an exhausted state from want of food, prolonged working hours, mental distress, &c., even in handicrafts not of themselves pernicious, is very detrimental to the organ. So that the later periods of work are those which are found most materially to weaken the sight and injure the eye. The shortening of working hours would probably be a saving in the end to both master and artisan; for the faulty execution of that which is completed with an imperfect organ must be a loss to the former, while the latter is ill remunerated by slightly increased wages for the risk of illness which he runs.

"11. The diffusion of knowledge bearing on the health of artisans by means of class journals, is worthy of the serious consideration of the philanthropist."

The committee reported also, that they had prepared a list of "desiderata" in the department of industrial pathology, for the exhibition of the Society—that is, of inventions required to protect the lives and health of working persons in various occupations; which list they recommended to be published and circulated.

They also recommended for special inquiry during the current year, as the basis of a report to be prepared by the ensuing January, either—

"The accidents which occur from the faulty construction of machinery, scaffolding, shipping, and other mechanical contrivances; or

"The injury to health arising from the inhalation of dust, grit, fluff, and similar foreign matters; or

"The effects of the exposure to damp and cold, rendered necessary by some employments."

*Journal of the Society of Arts.*

---

### ON THE TEACHING OF SANITARY SCIENCE IN MEDICAL AND PUBLIC SCHOOLS.

Great advance has been made in this direction during the past few months. The principles of hygiene and physiology are to be taught in Heriott's Hospital, Edinburgh. Chairs of military surgery and medicine are about to be established in all medical schools. A summer course of lectures on tropical medicine and hygiene, is to be delivered at St. Mary's Hospital, London, by Dr. James Bird, and a course on hygiene, in its general bearings, at the School of Anatomy and Medicine, adjoining St. George's Hospital, by Dr. Headlam Greenhow, whose long experience in sanitary matters will unquestionably render him a competent teacher of this important subject. We watch these signs of progress with great satisfaction.—The pity only is, that they appear so late.

---

### RETURN DUST IN MODEL LODGING HOUSES.

It may be considered as a fact perfectly certain, that there does not exist, either in the archives of architectural literature, or in any traditional knowledge exemplified in the achievements by which our cities are beautified and extended,—by which are raised edifices of varied grandeur and elegance, and buildings for every use, and of every grade, from the highest to the lowest class of habitable dwelling,—any sign indicative of defined views of the relative conditions of the atmosphere as it exists in its natural state, and as enclosed in dwellings and public buildings.

How entirely without reference to such knowledge, or without due attention to it, architectural works are often carried out, I will not now describe. My object is to notice certain contrivances, to be seen in more than one of the Model Lodging-houses of this metropolis, and which, though devised to accomplish good ends, produce little except inconvenience and mischief.

For the purpose of conveying away the cinders and other dry dirt and refuse of the apartments of each floor, there are formed in these buildings verticle shafts or flues, extending from the top to the basement. Each of these flues is made common to the use of all the several sets of apartments (which are over each other), and has a communication throughout, either by a flap-door opening up through the floor, or by a continuation through one of the walls. The flue is built over a dust-bin, likewise common to the series of floors, from the top to the bottom of the building.

Whenever the cinders and dust are thrown into this shaft, the lighter particles are forced back into the room, until the trap-door is again closed, and even when closed, the evil continues; for around the door or flap, as I have observed, light particles still intrude, so constantly, and in such quantity, as to produce accumulations of what has acquired amongst the inhabitants the name of "return dust," and which requires frequent sweeping away.

Now it is obvious that this is not the sole evil, and that exhalations, more or less impure, ascending from the refuse matter in the dust-bin, are carried with accelerated ascensional force up through the shaft to the several doors or flaps; and when it so happens that delay beyond the prescribed period for emptying the dust-bin occurs, or improper uses are made of the flue or of the dust-bin, then bad and injurious gases must ascend, and must be introduced into, and pervade, the atmosphere of the apartments.

In this example are seen not only the failure of a scheme which only indifferently acccomplishes the purpose for which it was intended (seeing that cleanliness was one of its objects), but an instance also of the creation of an evil, surpassing, in a sanitary point of view, all the good which would have been achieved, if every particle of the refuse matter poured into the flue had gone direct to the bin below, and "return dust" had never been guilty of half stifling, half blinding the inmates of the rooms. As the matter stands at present, "return dust" becomes a permanent commodity in the domestic economy of the place,—a still returning, never ending, pertinacious witness to an act of retrogression in sanitary science, which, in a "model house," can only be regarded as a model blunder.

---

### MORTALITY COMPARED WITH THE NUMBER OF MEDICAL PRACTITIONERS.

The question has been raised by M. Boudin, whether the increase of the number of medical practitioners has any influence upon the general mortality. He has endeavoured to answer that question by aid of the vital statistics of Prussia and Norway.

The subjoined table shews the general mortality in Prussia in those years in which the number of medical practitioners was subject to a census.

| Year. | Number of inhabitants to 1 medical practitioner. | Number of inhabitants to 1 death. |
|---|---|---|
| 1822 | 2,892 | 37·0 |
| 1825 | 2,955 | 37·4 |
| 1828 | 2,991 | 34·1 |
| 1831 | 3,305 | 28·1 |
| 1834 | 3,073 | 31·8 |
| 1837 | 2,928 | 32·1 |
| 1840 | 2,990 | 35·6 |
| 1843 | 2,876 | 34·8 |
| 1846 | 2,832 | 34·0 |
| 1849 | 2,787 | 32·7 |

It is evident from this table, that the proportion of the number of deaths to the number of inhabitants is far from having diminished with the increased number of medical practitioners. During the time when there was one death to 37 inhabitants, as in 1822 and 1825, there was only one medical practitioner to 2,900 inhabitants; while, in 1846 and 1849, there was one death to 33 living persons, notwithstanding that the number of medical men had increased to one in 2,800 inhabitants. It might, however, be objected (continued M. Boudin), that it is not merely the absolute number of medical practitioners, but their distribution, which affects the mortality of a country, assuming always that the professional competence of medical men remains the same in the various periods which are compared. M. Boudin compares the statistics of twenty-five of the larger Prussian towns, and gives a table confirming the result already arrived at for the whole of Prussia—that there is no relation whatsoever between an increase in the number of medical practitioners and the diminution of the proportion of deaths. For example, the towns of Coeslin and Münster have one and the same mortality—one death in 44 inhabitants; while the number of medical practitioners is at

| | |
|---|---|
| Coeslin . . . . . . | 1 in 5,118 inhabitants |
| Münster . . . . . . | 1 in 2,133 „ |

These facts prove anew the correctness of the statement of M. Ad. Quételet:* "L'art de guérir exerce peu d'influence sur le nombre des décès; mais il en a beaucoup pour améliorer physiquement le peuple. Il diminue la somme des douleurs, en même temps qu'il donne des consolations; cette mission est assez belle pour qu'on puisse ranger cet art parmi ceux qui servent le mieux d'humanité."—"The healing art exercises but little influence upon the number of deaths, but it contributes materially to ameliorate the physical condition of the people. It diminishes the sum total of pain, at the same time that it exerts its consoling influence. This is a noble mission, and entitles the medical profession to take rank among those arts which perform the greatest services to humanity."

We do not think it necessary to give an extract of the statistics of Norway, which were communicated to M. Boudin by Dr. Holst of Christiania, as they furnish the same results as we have shewn to be deducible from the statistics of Prussia. We have been surprised, indeed, to find such a question raised *at all*, as the one which M. Boudin has endeavoured to answer. It is evident that, as human life must of necessity come at some time to an end, the number of deaths must be determined by the number of persons living, and cannot be interfered with in any way whatever. Medicine could only change the proportion of deaths for a limited period in one way only—by prolonging the life of all or many persons to the ordinary period of old age. In this case, the number of deaths would diminish for several generations; but, after this, it would necessarily become the same as it was before. There is no doubt that already human life is *prolonged* in many instances, but not in so many as materially to alter the proportion of deaths; and as this progress, though decided, is of necessity slow, it could never be perceptible in statistics ranging over thirty years only. The statistics of the relation between the number of medical practitioners and the number of days of sickness would have given a very decided result in favour of the labours of the medical profession. It would have shewn that medical attendance shortens illness, and that the number of medical men stands in an inverted proportion with the number of days of disease.

---

### EMPLOYMENT OF THE STEAM-JET FOR THE CLEANSING OF SEWERS.

Mr. G. Gurney has reported to the Office of Works a new experiment, which he has tried with success, for removing or destroying the effluvia of sewers. The object is accomplished by means of the steam-jet, which produces a current through the sewer, and conveys with it the noxious exhalations, which are then decomposed and rendered harmless by being made to pass through a coke-fire as they are drawn out.

---

* Du Système Social et des Lois qui le Régissent. Paris: 1848. P. 191.

### APPLICATION OF THE SEWAGE OF TOWNS TO AGRICULTURAL PURPOSES.

This subject has lately been commented on in two instructive papers: one of which was read by Mr. Mechi, of Tiptree Hall, at a meeting of the London Farmers' Club on Feb. 5th; and another at an ordinary meeting of the Society of Arts on March 7th, by Mr. J. B. Lawes, F.R.S. These essays are, for the most part, of interest in relation to agriculture; but the subject is no less important in a sanitary point of view: The main object of the papers is to shew the importance of applying human ordure, in the liquid form, to the manuring of lands; and both papers contain a variety of interesting statistical calculations as to the amount which might be supplied from the population of London, but which is now entirely wasted.

---

### USE OF LIME-WATER IN THE FORMATION OF BREAD.

To neutralise the deterioration which the gluten of flour undergoes by keeping bakers add sulphate of copper or alum with the damaged flour. Professor Liebig, however, has conceived the idea of employing lime, in the state of solution, saturated without heat. After having kneaded the flour with water and lime, he adds the yeast, and leaves the dough to itself; the fermentation commences, and is developed as usual; and if we add the remainder of the flour to the fermented dough at the proper time, we obtain, after baking, an excellent, elastic, spongy bread, free from acid, of an agreeable taste, and which is preferred to all other bread after it has been eaten for some time. The proportions of flour and lime-water to be employed are in the ratio of 19 to 5. As the quantity of liquid is not sufficient for converting the flour into dough, it is completed with ordinary water. The quantity of lime contained in the bread is small—160 ounces of lime require more than 300 quarts of water for solution; the lime contained in the bread is scarcely as much as that contained in the seeds of leguminous plants. Professor Liebig remarks that "it may be regarded as a physiological truth, established by experiment, that corn flour is not a perfectly alimentary substance; administered alone, in the state of bread, it does not suffice for sustaining life. From all that we know, this insufficiency is owing to the want of lime, so necessary for the formation of the osseous system. The phosphoric acid likewise required is sufficiently represented in the corn, but lime is less abundant in it than in leguminous plants. This circumstance gives, perhaps, the key to many of the diseases which are observed among prisoners, as well as among children whose diet consists essentially of bread. . . . The yield of bread from flour kneaded with lime-water is more considerable. In my household, 19 pounds of flour, treated without lime-water, rarely give more than 24½ pounds of bread; kneaded with 5 quarts of lime-water, the same quantity of flour produces from 26 pounds 6 ounces to 26 pounds 10 ounces of well-baked bread. Now as, according to Heeren, 19 pounds of flour furnish only 24 pounds 1½ ounces of bread, it may be admitted that the lime-water bread has undergone a real augmentation."

*Annalen der Chemie und Pharmacie,* and *Chemist,* March 1855.

---

### MEANS OF PROCURING GOOD WATER IN CLAY DISTRICTS.

Mr. R. Varden, in the *Gardener's Chronicle* for January 13, recommends the collection of rain-water, by means of drainage pipes, from either arable or grass land, in clay districts, where the water of wells is generally bad. The water is kept in tanks, which, as the bad qualites of the well-water arise from the soluble matters in the clay with which it is in connexion, must be cemented so as to be water-tight. In many instances the existing well, if partially filled up so as to exclude the springs, might be lined and bottomed with cement at an expense of 2*l.* to 3*l.*, and made to answer the purpose. The size of the tank must, of course, be proportioned to the quantity of water required, and there must be a waste pipe from it.

---

### SUBSTITUTE FOR THE POTATO.

The employment of the Chinese yam (*Dioscorea Batatas*) as a substitute for the potato, has lately, we learn from the *Gardener's Chronicle* for Jan. 13, excited great interest in France. M. Decaisne, who has investigated the subject, "regards the Chinese yam as superior in quality to the potato. Although no comparative analysis of the two has been made, he believes that the Chinese yam is much the richer in point of nutritive principles. Its roots are as white as snow in the interior; they neither contain visible fibres nor tough woody matter; and when boiled, they become so soft that a slight pressure converts them into a paste, which can only be compared to that of the finest wheaten flour. Cooked by steam or roasted, they look and taste like the best potatoes. They have one advantage, which every one will appreciate, namely, the short space of time required for cooking. Two pieces of tubers, of the size of a hen's-egg, one the Chinese yam, the other the *Batata blanche*, were both put into boiling water at the same time with a Dutch potato of the same size; the first and second were done in ten minutes, the third in twenty minutes. The Chinese yam may be kept easily for a year, and perhaps longer. We all know that the potato is certain to sprout in spring. The Chinese yam is free from this disadvantage; it is neither affected by cold nor heat, and perhaps not even by moisture. Left in the ground, it remains alive throughout the winter without injury, as has been proved by a root which passed there the last severe winter, and pushed freely in spring."

M. E. Frémy (*Comptes Rendus*, January 15, 1855, and *Chemist*, March, 1855) has made an analysis of the Chinese yam, or *igname*. MM. Boussingault and Payen had also analysed the tubers of the plant, both as cultivated in France and in Algeria. The proximate principles are in great part the same as those of the potato. The yam yields only 16 per cent. of starch, while the potato furnishes 26 per cent.; but the yam contains a remarkable nitrogenous principle, which is not met with in the potato, and which may exert a beneficial influence in the uses of the tuber. The mucilaginous substance which communicates to the juice of the Dioscorea batatas unctuous properties, and which gives to the root when boiled a pasty consistence, is nitrogenous and coagulable by heat, but only after long boiling. Hence the Chinese yam, cut into small slices and dried in an oven, gives a product that may be reduced to powder, and which when treated with water, forms a paste resembling that of wheaten flour.

The *Gardener's Chronicle* for February 3, contains a review of *Remarks on the Tuberous Plants proposed as substitutes for the Potato*, by F. F. Mérat. The plants which it has been proposed to employ in this way have been the Dahlia, the *Boussingaultia baselloides*, the *apios tuberosa*, the *psoralea esculenta*, and the *lathyms tuberosus;* but of these, some cannot be employed on account of their nauseous taste, and others on account of their hard consistence. The Peruvian plant Ulluco may, M. Mérat thinks, possibly fulfil, though imperfectly, some of the conditions required. It much resembles the potato, but is liable to become hard by prolonged boiling. The possibility, then, of employing this plant as a substitute for the potato is very questionable.

---

### LAWS OF HEALTH.

It must be considered an established truth in science, that the health, the well-being, and the duration of life of men, are intimately connected with the observance of the natural laws of the universe in which he dwells. The acknowledgment of this fact is not, however, sufficient to insure obedience to these laws. Men must be brought individually and collectively to obey them,—each man for himself,—every family,—in order to insure its possession of that immunity from disease which the great Creator obviously intended his creatures to possess.—*Dr. Sutherland.*

